# The Impact of Anemia on the Survival of Patients Diagnosed With Low-Grade Malignant B-cell Lymphomas

**DOI:** 10.7759/cureus.65441

**Published:** 2024-07-26

**Authors:** Lidia-Maria Mondoc, Alina-Camelia Catana, Liiana-Carmen Prodan, Madalina Valeanu, Romeo-Gabriel Mihaila

**Affiliations:** 1 Hematology, Sibiu County Emergency Clinical Hospital, Sibiu, ROU; 2 Physiopathology, Faculty of Medicine, Lucian Blaga University of Sibiu, Sibiu, ROU; 3 Hematology, Faculty of Medicine, Lucian Blaga University of Sibiu, Sibiu, ROU; 4 Neurology, Sibiu County Emergency Clinical Hospital, Sibiu, ROU; 5 Biostatistics, Iuliu Hatieganu University of Medicine and Pharmacy, Cluj-Napoca, Cluj-Napoca, ROU

**Keywords:** hemoglobin, overall survival, prognostic factor, anemia, lymphoma

## Abstract

Introduction: B-cell lymphomas with a low degree of malignancy represent a heterogeneous group of diseases, that evolve slowly, but present particularities in terms of long-term survival.

Methods: We investigated the impact of anemia from the time of diagnosis in 249 patients with malignant B-cell lymphomas, diagnosed between January 2011 and December 2015, in the Hematology Department of the Sibiu County Emergency Hospital, Romania.

Results: We included 126 (50.6%) male and 123 (49.4%) female patients with the average age being 68.2 years. Among all patients, 106 (42.6%) were diagnosed with chronic lymphocytic leukemia (CLL), 61 (24.5%) with marginal zone lymphoma (MZL), 53 (21.3%) with multiple myeloma (MM), 16 (6.4%) with follicular lymphoma (FL), nine (3.6%) with plasmacytoma, and four cases with hairy cell leukemia (HCL). The serum Hb value in the subject group varied between 2.6 g/dL and 17 g/dL. At diagnosis, 18 (7.2%) patients had severe anemia, 32 (12.9%) had moderate anemia, 58 (23.3%) had mild anemia, and 141 (56.6%) had no anemia at all at the time of diagnosis. In our group, the higher degree of anemia was correlated with a more advanced stage of the disease but not with the older age of the patients. Our study's highest median value of LDH corresponded to moderate anemia and the lowest value to patients who did not have anemia. Patients who did not have anemia at diagnosis had the best survival at five years, followed by those with mild anemia, then those with moderate anemia.

Conclusion: In our cohort, subjects with the lowest Hb value at diagnosis had the worst survival. The results of our study conclude that anemia represents a negative impact factor not only on the patient's quality of life but also on their survival.

## Introduction

Anemia is the most common characteristic of B-lymphocyte lymphomas with a low grade of malignancy, encountered both at diagnosis and during cytotoxic treatment, as a complication of the lymphomas [1]. According to the WHO, anemia is defined as the absolute reduction of the level of hemoglobin (Hb or HB) in the circulating blood below the reference values for the patient's age, sex, and physiological state (Hb < 13 g/dL in men and Hb < 12 g/dL in women). Although the role of the anemic syndrome in cancers was the subject of medical research started many years ago, its pathogenesis and impact in the evolution of chronic lymphoproliferations remains an open topic and of interest to the medical world even today.

It seems that anemia is the result of a combination of factors [2], such as the involvement of the bone marrow in which iron is abnormally reused [3], abnormal metabolism of iron in malignant and chronic inflammatory diseases [4], the inadequate response of the bone marrow to the action of erythropoietin [5,6], and the inhibition of erythropoiesis by certain inflammatory mediators such as TNF, IL-1, IL-10 [7-9], IL-6 [7,10] and interferon γ [11].

An association has been observed between disease activity and the release of IL-6 into the circulatory system, stimulating the overproduction of hepcidin. The increased level of serum hepcidin causes the capture of iron in macrophages and enterocytes and blocks the release of iron from the reticuloendothelial system and liver [2,12].

Other possible causes of anemia occurring in malignant hematological diseases can be nutritional deficiencies, shortening of the lifespan of erythrocytes, extracorpuscular hemolysis, dislocation of normal bone marrow by tumor tissue, amyloid deposits in the marrow, bone marrow necrosis, autoimmune hemolytic anemia, and blood loss [13]. In addition to all this, anemia can be accentuated by cytotoxic treatments, so the relationship between anemic syndrome and malignant hemopathies is complex and still incompletely known.

However, several studies from the last decades have revealed a common thing: intratumoral hypoxia is responsible for the much more aggressive nature of tumor cells, and can even negatively influence the response to antitumor treatment [14-16]. Moreover, the persistence of anemic syndrome after chemotherapy in patients with malignant lymphomas is strongly associated with a higher risk of disease relapse [17]. Other studies reported the incidence and the impact of anemia on bone marrow in patients with malignant non-Hodgkin's lymphoma, but only a few have analyzed anemia at diagnosis, as a prognostic factor for a patient's overall survival [18].

In this article, we analyzed the value of Hb at diagnosis as a prognostic factor regarding the overall survival of patients diagnosed with malignant B-cell non-Hodgkin lymphoma with a low grade of malignancy.

## Materials and methods

A five-year retrospective study was undertaken at the Hematology Department of the Sibiu County Emergency Clinical Hospital, Sibiu, Romania, between January 2011 and December 2015. The study involved a total number of 249 patients who were diagnosed with the following types of malignant lymphomas: chronic lymphocytic leukemia (CLL), multiple myeloma (MM), plasmacytoma, hairy cell leukemia (HCL), follicular lymphoma (grade 1 and 2) (FL) and marginal zone lymphoma (MZL). 

Those patients who had available data regarding the Hb value at the time of diagnosis, the result of lymph node and bone marrow biopsies, and the result of imaging examinations necessary for staging, were included in the study. Those who did not previously receive blood transfusions, chemotherapy, radiotherapy, or immunotherapy were also included in the study.

Patients without a histopathological certainty diagnosis of lymphoma, those whose Hb value was not found at the time of diagnosis, and patients diagnosed with grade 3 follicular lymphoma were excluded from the study. Patients who received previous treatment for anemic syndrome were also excluded from the study.

The histopathological diagnosis of the types of lymphomas that were included in this study was established according to the 2016 WHO classification of tumors of hematopoietic and lymphoid tissue [19]. The following disease stagings were used: Binet staging for CLL, Durie-Salmon staging for MM, and Ann Arbor staging for FL and MZL.

We classified anemia in the following grades of severity according to the National Cancer Institute: mild (Hb level above 10.0 g/dL), moderate (Hb level 8.0 to 10.0 g/dL), and severe (Hb level below 8.0 g/dL) [20].

For statistical analysis and data description, IBM SPSS Statistics for Windows, Version 25, (Released 2017; IBM Corp., Armonk, New York, United States) was utilized. A statistical significance threshold of α = 0.05 was established. For the description of normally distributed continuous quantitative data, the mean (M) ± standard deviation (SD) was utilized, and the median (first quartile-third quartile) for non-normally distributed quantitative data was utilized. Time variables were described using the median. The absolute and relative frequencies (%) were employed for the description of qualitative data.

The Student t-test was utilized to compare the means of two independent groups' normally distributed data. The means of two independent groups with non-normal distributions were compared using the non-parametric Mann-Whitney and Kruskal-Wallis tests. Fisher or chi-square tests were used to compare qualitative variables. The Kaplan-Meier test and survival curves were used to compare the survival time between different groups.

## Results

A total of 249 patients, with an M age=68.2 (SD=10.9) and a balanced number of men and women (50.6%/49.4%), were included in the study. Among all the patients, 42.6% (106) had CLL, 24.5% (61) had MZL, 21.3% (53) had MM, 6.4% (16) had FL, 3.6% (nine) had plasmacytoma, and there were four cases (1.6%) with HCL. The sample consisted of 126 (50.6%) men and 123 (49.4%) women aged between 25 and 93 years old, the average age being 68.2 years with an SD of 10.98 years.

The Hb levels for the whole group of patients ranged from 2.6 g/dL to 17.0 g/dL, with an average of M=12.00 (SD=2.42). Severe anemia was encountered in 7.2% (18) of the cases, moderate anemia in 12.9% (32), mild anemia in 23.3% (58), and 56.6% (141) did not have anemia. The median and degree levels of Hb for each lymphoma type and subtype are shown in Table 1. 

**Table 1:** The distribution of the grades of anemia encountered in the studied group of patients.

Type of lymphoproliferation	N (%)	Hb value	
		Mean±standard deviation	Median (interquartile range)
Chronic lymphocytic leukemia	106 (42.6)	12.85±2.14	13 (11.80;14.40)
Multiple myeloma	53 (21.3)	9.78±2.08	9.8 (7.90;11.40)
Hairy cell leukemia	4 (1.6)	11.98±3.77	11.5 (8.85;15.10)
Plasmacytoma	9 (3.6)	13.93±2.54	14.2 (12.00;16.30)
Follicular lymphoma	16 (6.4)	12.53±1.26	12.65 (11.90;13.70)
Marginal zone lymphoma	61 (24.5)	12.11±2.11	12.55 (10.80;13.50)

**Figure 1:**
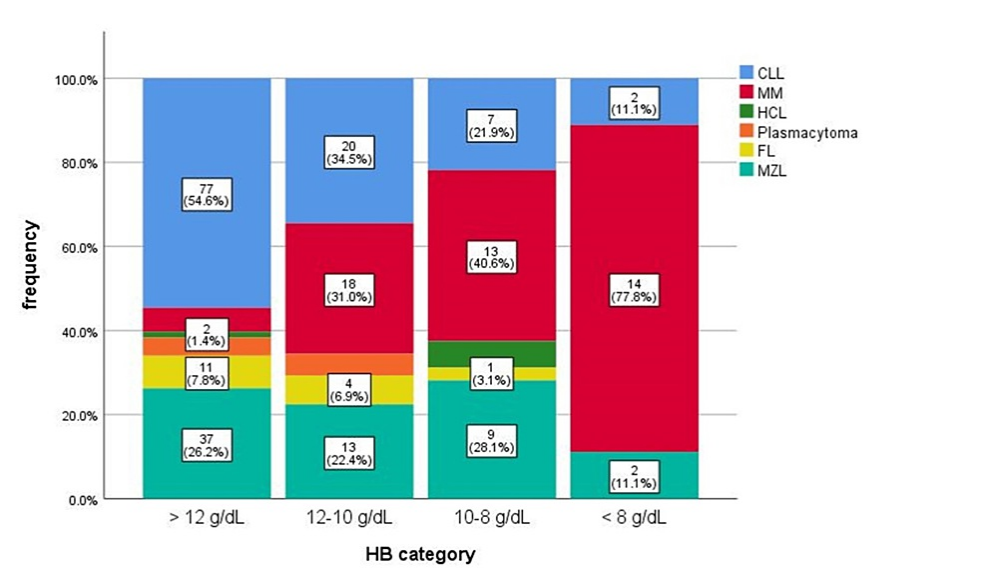
Chronic lymphoproliferations depending on anemia degree. CLL: chronic lymphocytic leukemia; MM: multiple myeloma; HCL: hairy cell leukemia; FL: follicular lymphoma; MZL: marginal zone lymphoma

The distribution of chronic lymphoproliferation according to the degree of anemia can be seen in Figure 1.

There were significant differences between the median Hb values of the lymphoproliferation types (p<0.001). The post-hoc analysis carried out using the Bonferroni test showed that there were significant differences between the median Hb values in MM type (median: 9.8; IQR: 7.9 - 11.4) ​​and median Hb levels in MZL (median: 12.5; IQR: 10.8 - 13.5); p<0.001, FL (median: 12.6; IQR: 11.9 - 13.7); p<0.001, CLL (median: 13.0; IQR: 11.8 - 14.4); p<0.001, and plasmacytoma (median: 14.2; IQR: 12.0 - 16.3); p<0.001. 

The distribution of anemia according to disease stages is presented in Table 2.

**Table 2:** The distribution of anemia according to disease stages. CLL: chronic lymphocytic leukemia; MM: multiple myeloma; HCL: hairy cell leukemia; FL: follicular lymphoma; MZL: marginal zone lymphoma; MGUS: monoclonal gammopathy of undetermined significance

		N(%)	HB (g/dL) Median (IQR)	HB (g/dL)			
				>12 N(%)	10-12 N(%)	8-10 N(%)	<8 N(%)
CLL		106(42.6)	13.0(11.8;14.4)	77(54.6)	20(34.5)	7(21.9)	2(11.1)
CLL Binet staging	A	29(27.4)	14.0(12.8;15.0)	27(93.1)	2(6.9)	-	-
	B	56(52.8)	13.0(12.0;14.4)	41(73.2)	12(21.4)	3(5.4)	-
	C	21(19.8)	11.1(9.9;13.3)	9(42.9)	6(28.6)	4(19.0)	2(9.5)
MM		53(21.3)	9.8(7.9;11.4)	8(15.1)	18(34.0)	13(24.5)	14(26.4)
MM Durie-Salmon staging	I	8(15.1)	10.3(9.9;11.9)	1(12.5)	5(62.5)	1(12.5)	1(12.5)
	II	10(18.9)	9.1(7.9;9.5)	-	2(20.0)	5(50.0)	3(30.0)
	III	33(62.2)	9.8(7.7;11.4)	5(15.2)	11(33.3)	7(21.2)	10(30.3)
	MGUS	2(3.8)	12.7(12.3;13.0)	2(100.0)	-	-	-
MM substages-A/B	A	33(62.2)	10.0(8.3;11.5)	5(15.2)	12(36.4)	9(27.3)	7(21.2)
	B	18(34.0)	9.4(7.5;10.5)	1(5.6)	6(33.3)	4(22.2)	7(38.9)
	MGUS	2(3.8)	12.7(12.3;13.0)	2(100.0)	-	-	-
HCL		4(1.6)	11.5(8.9;15.1)	2(50.0)	-	2(50.0)	-
Plasmacytoma		9(3.6)	14.2(12.0;16.3)	6(66.7)	3(33.3))	-	-
FL		16(6.4)	12.7(11.9;13.7)	11(68.8)	4(25.0)	1(6.2)	-
FL stages	I	2(12.4)	14.0(14.0;14.0)	2(100.0)	-	-	-
	II	4(25.0)	12.9(12.4;13.5)	3(75.0)	1(25.0)	-	-
	III	3(18.8)	12.1(11.9;12.6)	2(66.7)	1(33.3)	-	-
	IV	7(43.8)	12.2(10.6;13.6)	4(57.1)	2(28.6)	1(14.3)	-
MZL		61(24.5)	12.5(10.8;13.4)	37(60.7)	13(21.3)	9(14.8)	2(3.3)
MZL stages	I	10(16.3)	14.0(14.0;14.0)	8(80)	2(20)	-	-
	II	8(13.12)	12.9(12.4;13.5)	8(100)	-	-	-
	III	4(6.56)	12.1(11.9;12.6)	2(50)	1(25)	1(25)	-
	IV	39(63.93)	12.2(10.6;13.6)	19(48.7)	10(25.6)	8(20.5)	2(5.1)

There were no significant differences between the median Hb values ​​in women (12.4 (IQR: 10.7-13.3)) compared to men (12.4 (IQR: 10.28-14.2)), p=0.241. The M Hb values ​​were significantly higher in men (15.92) than in women (11.45) only for patients with plasmacytoma (p=0.016). For the other types of lymphoproliferations, there were no significant differences between the sexes. The incidence of anemia depending on gender is shown in Table 3.

**Table 3:** The occurrence of anemia depends on the gender of the patient. (*) Chi-square or Fisher's exact test was used to check the association between gender and HB groups: >12 g/dL, 10-12 g/dL, 8-10 g/dL,<8 g/dL. NS=not significant, p≥0.05. CLL: chronic lymphocytic leukemia; MM: multiple myeloma; HCL: hairy cell leukemia; FL: follicular lymphoma; MZL: marginal zone lymphoma

Gender (M/F)		HB (g/dL)				
	Overall N(%)	>12 N(%)	10-12 N(%)	8-10 N(%)	<8 N(%)	p-value*
CLL						
M	58(54.72%)	35(72.92)	10(20.83)	3(6.25)	-	NS
F	48(45.28%)	42(72.41)	10(17.24)	4(6.9)	2(3.45)	
MM						
M	25(47.17%)	6(21.43)	10(35.71)	6(21.43)	6(21.43)	NS
F	28(52.83%)	2(8)	8(32)	7(28)	8(32)	
HCL						
M	3(75%)	1(100)	-	0(0)	-	NS
F	1(25%)	1(33.33)	-	2(66.67)	-	
Plasmacytoma						
M	5(55.56%)	1(25)	3(75)	-	-	0.048
F	4(44.44%)	5(100)	0(0)	-	-	
FL						
M	5(31.25%)	8(72.73)	2(18.18)	1(9.09)	-	NS
F	11(68.75%)	3(60)	2(40)	0(0)	-	
MZL						
M	30(49.18%)	19(61.29)	6(19.35)	5(16.13)	1(3.23)	NS
F	31(50.82%)	18(60)	7(23.33)	4(13.33)	1(3.33)	

The incidence of anemia depending on age is shown in Table 4. In the case of CLL, patients aged >60 had mean Hb values ​​(14.75 g/dL) significantly higher than the others (12.9 g/dL) (p<0.001). For the other types of lymphoproliferations, the differences between the average values ​​of Hb by age categories did not differ statistically significantly. There were 50 (20.08%) patients aged >60 and 199 (79.92%) aged ≤60. Patients aged >60 years had significantly higher Hb values (M=12.84 (SD=2.54)) ​​than the others (M=11.78 (SD=2.36)), p=0.004.

**Table 4:** The occurrence of anemia depends on the age of the patient. P-values indicate the level of statistical significance for the association between age and HB levels. NS=not significant, p≥0.05; (*) Chi-square or Fisher's exact test was used to check the association between age group (>60 years versus ≤60 years) and HB groups: >12 g/dL, 10-12 g/dL, 8-10 g/dL,<8 g/dL. CLL: chronic lymphocytic leukemia; MM: multiple myeloma; HCL: hairy cell leukemia; FL: follicular lymphoma; MZL: marginal zone lymphoma

Age (years)		HB (g/dL)				
	Overall	>12 N(%)	10-12 N(%)	8-10 N(%)	<8 N(%)	p-value*
CLL						
>60	22(20.75%)	19(86.36)	3(13.64)	-	-	NS
≤60	84(79.25%)	58(69.05)	17(20.24)	7(8.33)	2(2.38)	
MM						
>60	9(16.98%)	1(11.11)	4(44.44)	2(22.22)	2(22.22)	NS
≤60	44(83.02%)	7(15.91)	14(31.82)	11(25)	12(27.27)	
HCL						
>60	1(25%)	0(0)	-	1(100)	-	NS
≤60	3(75%)	2(66.67)	-	1(33.33)	-	
Plasmacytoma						
>60	4(44.44%)	2(50)	2(50)	-	-	NS
≤60	5(55.56%)	4(80)	1(20)	-	-	
FL						
>60	4(25%)	3(75)	1(25)	0(0)	-	NS
≤60	12(75%)	8(66.7)	3(25)	1(8.3)	-	
MZL						
>60	10(16.39%)	7(70)	2(20)	1(10)	0(0)	NS
≤60	51(83.61%)	30(58.82)	11(21.57)	8(15.69)	2(3.92)	

**Figure 2:**
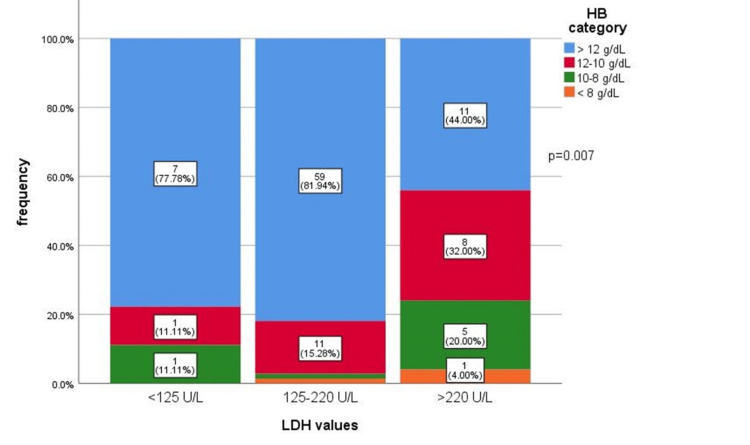
Distribution of HB categories according to LDH for patients with chronic lymphocytic leukemia. A chi-square test was used.

Anemia is statistically significantly associated with LDH classes for patients with CLL (p=0.007) (Figure 2).

LDH has a significantly higher median value in MZL (265) compared to CLL (219) and FL (249), the frequency of high values ​​(36%) being significantly higher compared to the other lymphoproliferations (Table 5).

**Table 5:** LDH category according to chronic lymphoproliferations. (*) A chi-square test was used. CLL: chronic lymphocytic leukemia; FL: follicular lymphoma; MZL: marginal zone lymphoma

Lymphoproliferation	LDH (U/L)			
	<125 U/L N(%)	125-220 U/L N(%)	>220 U/L N(%)	p-value*
CLL	9(8.49)	72(67.92)	25(23.58)	<0.001
FL	1(6.25)	11(68.75)	4(25)	
MZL	4(6.56)	35(57.38)	22(36.07)	

There were significant differences between LDH medians for the anemia categories (p=0.001). The highest LDH median value corresponded to the group of patients with HB<8 g/dL (285.5), and the lowest to those with HB>12 g/dL. Depending on the lymphoproliferation, a higher M HB was found in the case of MZL (265g/dL).

For patients with CLL, there were significant differences between LDH values ​​according to Hb classes (p=0.006). The highest median value corresponded to class 8-10 of Hb, and the lowest to class >12 Hb (Table 6).

**Table 6:** Correlation of the serum LDH value with the degree of anemia. P-values indicate the level of statistical significance for the association between Hb levels and lymphoproliferation. NS=not significant, p≥0.05; (*) Kruskal Wallis test was used. CLL: chronic lymphocytic leukemia; FL: follicular lymphoma; MZL: marginal zone lymphoma

LDH (U/L)	Hb>12 g/dL: Median (IQR)	Hb 10-12 g/dL: Median (IQR)	Hb 8-10 g/dL: Median (IQR)	p-value*
CLL	177 (159-204)	208.5 (155-246.5)	286 (209-374)	0.006
FL	181 (146-207)	205.5 (158.25-254.25)	-	NS
MZL	205 (165-235.5)	213 (159-309)	250 (194.5-328)	NS

Related to the disease risk of CLL, there was a statistically significant association between the Binet stages and the Hb categories (Table 7). Stages with a high risk of the disease (stage C) appear significantly more often in patients with low Hb values. 

**Table 7:** Incidence of anemia according to CLL stage. (*) A chi-square test was used. CLL: chronic lymphocytic leukemia

CLL-Binett Stage	Overall	HB (g/dL)				
	N(%)	>12 N(%)	10-12 N(%)	8-10 N(%)	<8 N(%)	p-value*
A	29(27.36)	27(0.93)	2(0.07)	-	-	<0.001
B	56(52.83)	41(0.73)	12(0.21)	3(0.05)	-	
C	21(19.81)	9(0.43)	6(0.29)	4(0.19)	2(0.1)	

**Figure 3:**
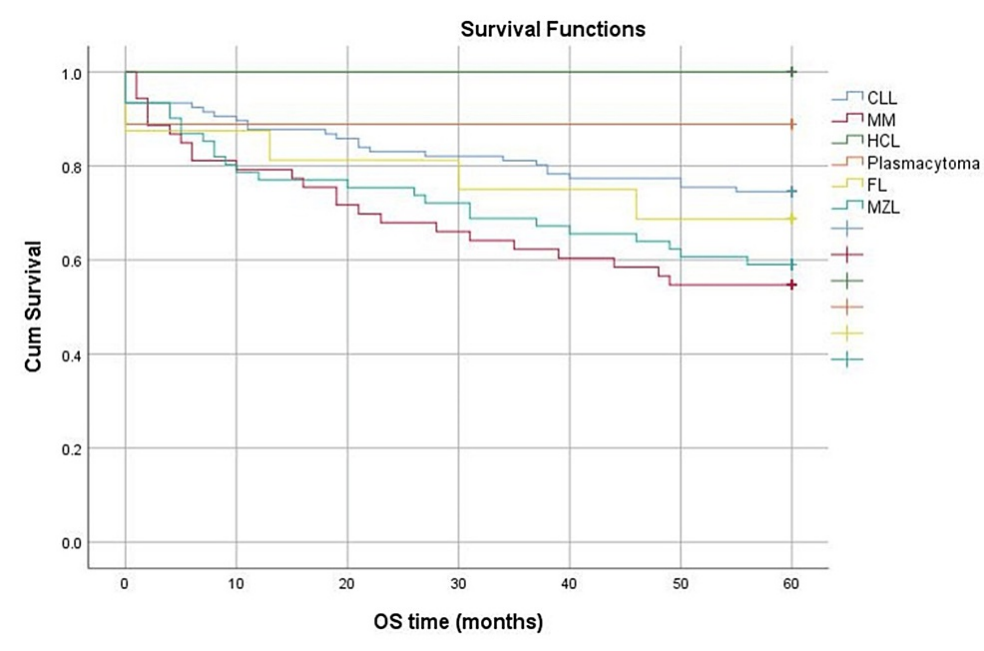
Survival at five years depending on the type of lymphoproliferation. CLL: chronic lymphocytic leukemia; MM: multiple myeloma; HCL: hairy cell leukemia; FL: follicular lymphoma; MZL: marginal zone lymphoma

Patients with CLL have the best survival, followed by those with plasmocytoma, and those with MM have the worst survival (p=0.041) Figure 3. The median survival time is 60 months for all types of lymphoproliferation.

**Figure 4:**
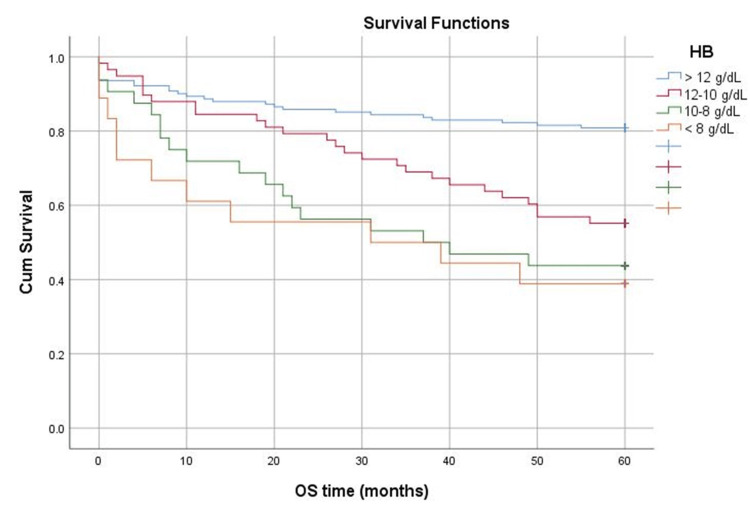
Survival of patients depending on the degree of anemia. OS: overall survival

Age did not significantly influence survival time (p=0.182). Patients with Hb >12 g/dL had the best survival, followed by those with Hb within 10-12 g/dL, then 8-10 g/dL. Those with the lowest Hb had the worst survival (p<0.001) (Figure 4). The median survival time for Hb >12 g/dL and Hb within 10-12 g/dL was 60 months; for Hb within 8-10 g/dL, it was 38.5 months; and for Hb <8 g/dL, it was 35 months.

## Discussion

Anemia is the most common characteristic in patients diagnosed with cancer, but also the most persistently affected biological parameter that they have to face, sometimes even for a long period of time after the end of antineoplastic treatment. Data from the literature show that in patients with malignant hemopathies, anemia can be considered an important prognostic factor regarding the evolution of the disease, which not only influences the survival of these patients but also significantly reduces their quality of life [13].

The current five-year retrospective study (January 2011-December 2015) included 249 patients diagnosed with the following malignant non-Hodgkin's lymphomas with a low grade of malignancy: CLL (42.6%), MM (21.3%), plasmacytoma (3.6%), HCL (1.6%), FL grade 1 and 2 (6.4%) and MZL (24.5%). 

In this study, the incidence of lymphoma was higher in men (50.6%) compared to women (49.4%), which correlates with the official data of the National Cancer Institute (Surveillance, Epidemiology, and End Results (SEER) program) [21], but also with a study conducted by Bukhari et al., which reported a higher incidence of lymphoma among men (78.79%), compared to women (21.21%) [22].

Recent data from the literature show a higher incidence of lymphomas with increasing age, especially after 60 years [21,23]. In our study, most patients (79.92%) were up to 60 years old, but the patients over 60 years of age presented significantly higher values ​​of the Hb level compared to the others.

The prevalence of anemia in the present study was 43.4%. Similar results were reported in other studies, where anemia was found between 32% and 49% [24].

According to the classification of the National Cancer Institute [20], we found that 7.2% of the subjects included in the study had severe anemia at diagnosis, 12.9% moderate anemia, and 23.3% mild anemia. A total of 56.6% of the patients in the studied group did not have anemia at the time of the diagnosis of malignant non-Hodgkin's lymphoma.

Interestingly, we did not find significant differences regarding the incidence of anemia according to gender. Some studies have explained this result due to menstrual blood loss in women who are still at reproductive age [13]. Even more, a significant association between male gender and higher levels of hepcidin and IL-6 was confirmed by Tisi et al. [6], which was also confirmed by another study led by Hohaus [12], who pointed out that there were higher levels of hepcidin in men compared to women. This would mean that anemia should occur more frequently among men compared to women, a result not confirmed in our study. However, our result can be debated due to the relatively small number of subjects included in the study.

We analyzed the degree of anemia encountered in each type of chronic lymphoproliferation that was studied. Thus, mild anemia was found in CLL in a percentage of 34.48%, in MM, the percentage was 31.03%, in MZL 22.41%, while in Plasmacytoma and FL, mild anemia was observed in less than 10% percentages. Moderate anemia was suffered by 21.88% of patients with CLL, 40.63% of those with MM, 28.13% of those with MZL, and 6.25% of patients with HCL. Most patients with severe anemia (77.78%) were diagnosed with MM, followed by those with CLL and MZL (11.11%). It was interesting to see that the most patients who did not present anemia at all at diagnosis were those with CLL, while in MM the most cases of severe anemia were registered. The result of our study showed significant differences between the median values ​​of Hb between the types of lymphoproliferations (p<0.001).

In the current study, we also wanted to analyze the value and impact of LDH in correlation with the level of anemia in patients with malignant non-Hodgkin's lymphomas. We analyzed the LDH value in patients with CLL, MZL, and FL. We thus found a significantly higher median LDH value in MZL compared to CLL and FL.

An interesting finding was that for the patients with CLL, significant differences were observed between the LDH value according to the degree of anemia (p=0.006). The highest median value of LDH in our study corresponded to moderate anemia (Hb 8-10 g/dL), and the lowest value to the class of Hb >12 g/dL, of those patients who did not present any anemia at the time of diagnosis. This confirms older data from the literature as well as the most recent ones, in which a significant association of anemia with elevated serum LDH levels was observed [25,26]. A possible explanation would be that patients with a higher level of LDH also have a higher concentration of IL-10, which stimulates increased production of hepcidin, involved in the pathogenesis of anemia from chronic diseases [7].

We also found in the present study a statistically significant association (p<0.001) between disease risk for CLL and serum Hb level. The lowest Hb values ​​were found in patients with high disease risk (Binet stage C). This could represent an interesting topic of debate regarding the risk factors of disease progression in CLL. Genetic, molecular, demographic, and clinical factors and the International Prognostic Index for patients with CLL (CLL-IPI) score are already accepted and known. According to the data obtained in our study, it would be useful to consider the Hb value at the time of diagnosis as a risk factor for disease progression. This could be a simple, cheap method and available to any CLL care center.

We analyzed survival at five years from diagnosis in our cohort, and patients diagnosed with CLL had the best survival, followed by those with plasmacytoma, while those with MM had the worst survival.

Age did not significantly influence the survival time, but the Hb level did. Patients who did not have anemia at diagnosis had the best survival, followed by those with Hb between 10-12 g/dL, and then those with Hb between 8-10 g/dL. Subjects with the lowest Hb level had the worst survival (p<0.001). The average survival time for patients who presented severe anemia at diagnosis (Hb < 8 g/dL) was 35 months, almost half less than the established follow-up period in the study (60 months). This fact aligns with the results of many similar studies [18,24,27], which concluded that anemia has a negative impact not only on the patient's quality of life and response to treatment but also on their long-term survival.

The limitations of our study are related to the relatively small number of subjects included compared to other studies, and to the retrospective nature of the cohort, which forced us to exclude a consistent number of subjects due to uncertain and incomplete data. Another limitation is that the study was conducted in a single center, which could prevent the generalization of the results to a wider patient population. Also, the study of the impact of anemia only on patients diagnosed with B-cell lymphomas with a low degree of malignancy may represent another limitation of the present study. Additional studies are needed, including those on patients with malignant hemopathies with a more aggressive character, such as acute leukemias or large B-cell lymphoma, in order to analyze the impact of anemia in these types of hematological cancer, which have very different characteristics.

## Conclusions

This retrospective study analyzed the importance of the level of serum Hb at the time of diagnosis and the impact of anemia in terms of survival in patients diagnosed with low-grade B-cell lymphomas. Patients who presented the lowest Hb value at the time of diagnosis had the worst survival at five years (with an average duration of 35 months), and patients with Hb > 12 g/dL had the best. These results are similar to other studies. Anemia and its degree have a negative impact on survival among such patients.
